# Challenges to court room mental health decision-making in the face of uncertainty

**DOI:** 10.3389/fpsyt.2026.1767080

**Published:** 2026-04-02

**Authors:** Conor Duggan, Jaydip Sarkar

**Affiliations:** 1 Division of Psychiatry and Applied Psychology, University of Nottingham, Nottingham, United Kingdom; 2 Sarkar Mental Health Experts Pty Ltd, Melbourne, VIC, Australia

**Keywords:** courtroom, expert psychiatric evidence, solutions, translating group to individual data, violence

## Abstract

Mental health professionals are frequently faced with the challenge of not only having to make decisions that have far-reaching consequences for the individual being assessed, but having to do so with inevitable degrees of uncertainty. Acknowledging and attempting to quantify this uncertainty ought therefore to be central to such an endeavour but, we argue, it is insufficiently recognised by the professionals involved. Specifically, ‘experts’ who rely on scientific evidence in providing an opinion need to be aware of the challenges of applying such group data to an individual case. In this paper, first, we consider some criticisms of current practice in this respect. Second, we offer a rejoinder that considers the place of tacit knowledge in the clinical practice, the contribution of both Bayesian reasoning and an adapted epidemiological criteria for causal inference, all of which we believe would strengthen the provision of a forensic ‘expert’ opinion, in a courtroom setting.

## 1 Introduction

“We thus need to distinguish between how well a model may predict for groups of patients, and how well for individual patients. …We believe that the distinction between what is available at a group and individual levels are not well understood.” (1, p. 454).

There are two reasons why the provision of expert evidence by mental health practitioners in court now merits further discussion. First is the belief that clinical practice in general, and its legal application specifically, ought to be evidence-based (2, 3). In the prediction of future violence among mentally disordered individuals, for instance, a number of actuarial instruments have been developed that have superior predictive validity as compared with a clinical opinion Yang et al. (4). In as much as such data relies on that derived from a group rather than from an individual, there is the challenge of applying such group data to an individual circumstance Scurich et al. (5). The current provision of expert opinion has drawn criticism in particular from Faigman et al. (6) and Dawid et al. (7) that we discuss below. Second, we discuss the different expectations from legal and clinical perspective that the court requires from expect witnesses that has been highlighted in a case in the UK (8). Finally, we offer a number of possible solutions that might resolve these issues.

In this paper, we wish to highlight some of the uncertainty that inevitably underpins such evidence as offered by experts. Specifically, society identifies ‘experts’ as being able to provide more valued estimates on the matter in question as compared to the ordinary individual. But, what does this comprise? Knowledge and experience are obvious criteria. However, there is also a reliance on ‘science’ as the latter is considered more objective than mere opinion.

Although few would argue against the desirability of basing clinical decisions and their legal implications on best available scientific evidence, there are problems with its implementation in practice. First, the relevant research evidence may be unavailable or be of such poor quality that the wrong conclusions are drawn; for example, from underpowered studies with small effect sizes which are prone to bias (9). Second, and more importantly for our argument here, there is the challenge of applying even good research ‘evidence’ - that inevitably comes from population or group data - to an individual case.

## 2 Evidence-based medicine and the challenge of its application to individuals

The wide-spread acceptance of ‘evidence-based medicine’ (10) with its dependence on evidence from group-based data has resulted in a challenge of how such data can be applied to an individual. Clearly, these decisions ought to follow from the combination of best evidence on the intervention together with the features of the individual case. We argue that, this integration of the evidence is largely intuitive, based on the training and experience of the clinician involved, rather than calculated in a way that can be made explicit. The same challenge faces the expert when marshalling evidence in court proceedings – a proposition which is further developed below (See Section on Tacit Knowledge).

## 3 Mental health evidence in courts

“Whereas scientists almost invariably inquire into phenomena at the group level, trial judges typically need to resolve cases at an individual level.” (6, p. 417).

We will focus here on providing mental health evidence for violence in a legal context. If one puts to one side, whether or not the individual is fit to plead, it is possible to reduce the questions asked of mental health experts in criminal courts to two core issues:

(a) X, with a history of a mental disorder has been accused of a (usually violent) offence. The question here seeks a causal explanation: to what extent has X’s mental disorder played a role in the offence?

(b) X, with a mental disorder, has been convicted of a violent or sexual offence is now facing release. Here, the question is about prediction: Will s/he do it again?

We shall return to this crucial distinction between what Dawid et al. (11) describe as ‘back-casting’, as in (a), and forecasting, as in (b). The challenge for the expert in court in both situations is that they are drawing upon group data, while dealing with a singular occurrence, when asked to provide an opinion on “questions of who did what, where, how and why, with what motive or intent’ (12) cited in 6).

## 4 Framework versus diagnostic evidence

Faigman et al. (6) propose a distinction between framework evidence and diagnostic evidence in laying down admissibility standards for expert testimony. The former refers to the description of general scientific propositions (group data based) and the latter to the application of these to an individual case (extrapolating to the individual case). Taking an example in forensic mental health, ‘framework evidence’ would refer to the prevalence of violence in people diagnosed with schizophrenia whereas ‘diagnostic evidence’ would refer to the likelihood that in someone diagnosed with schizophrenia, this caused/contributed to the individual’s violent behaviour.

According to Faigman et al. (6), this distinction between framework and diagnostic evidence has implications for how an expert witness’s evidence ought to be treated by the Court. Indeed, the bulk of their paper (pp. 440-480) seeks to lay down best practice guidelines on how these two types of evidence ought to be evaluated against the criteria of its (a) relevance, (b) qualifications (of the experts), (c) scientific validity, (d) added value, and (e) unfair prejudice. Here, we will focus on qualifications and scientific validity, which are especially relevant to this article.

Regarding qualifications, these authors emphasise that, of the ‘knowledge, skills and experience’ triad, for the diagnostic (as opposed to the framework) expert; greater emphasis ought to be placed on the ‘skills and experience’ in addition to the ‘knowledge’ of the expert. They also stress the importance here of ‘hands on experience’ in evaluating and treating people who are mentally ill and charged with criminal offences and to have the skill in gathering information ‘…that non-experts, or even framework experts, would find [it] difficult to obtain.’ (p. 447).

Regarding scientific validity in the US, they refer to the Frye and Daubert tests (13, 14), which set out criteria whereby expert evidence is or is not admissible, with judges having the obligation to assess whether the expert’s theory or technique: (1) is testable, (2) has adequate standards to control its operation, (3) has an acceptable and known error rate, (4) is subjected to peer review and publication, and (5) has “widespread acceptance” in the scientific community.

These are indeed demanding standards for an expert to meet, and there is empirical evidence that these ideals are not realised in practice. Judges, for instance, who are not conversant in a particular field have difficulty when deciding on the admissibility of evidence when faced with dissimilar conclusions proffered by opposing experts (15). Faigman et al. (6) also recognise that it is difficult for a diagnostic expert to verify scientifically his/her opinion in providing even framework evidence for a variety of reasons (pp. 455 et seq.). For instance, information on error rates may not be available, there may be publication bias etc. While they suggest as an antidote that the diagnostic expert follow an accepted protocol (p. 456); yet here again, we believe that the authors may be a little naïve in believing that such protocols have a firm scientific foundation as in many cases they are no more than accepted expert opinion – this being the lowest in the hierarchy of the evidence pyramid. More importantly, these considerations focus on the provision of framework evidence and do not address the application of such evidence to an individual case (i.e. diagnostic evidence).

In summary, while the authors are to be commended for distinguishing group (framework) from individual (diagnostic) evidence, and proposing criteria by which each might be evaluated, in our view, the practical application of the principles to providing ‘diagnostic’ evidence in the case of the individual remains problematic.

## 5 ‘Back-casting’ or forecasting

There is an important distinction between providing: (a) an explanation as to why something occurred in the past – for example, positing a ‘causal link’ between characteristics of a diagnosed mental disorder and a past specified offence, and (b) providing a risk assessment on the likelihood that someone will behave violently in the future; Dawid, et al. (11) refer to these as ‘back-casting’ and forecasting respectively and have an especial resonance in a forensic setting.

In the case of ‘back-casting’, an untoward event has already occurred and the task is to explain its occurrence. A patient with a mental disorder has committed a violent act; has the presence of the mental disorder contributed to the violent act? Similarly, a child presents to an accident and emergency department, for example, with an injury and the question is whether this injury is a sign of child abuse?

In forecasting, the focus is in the future. For instance, a decision has to be made in child protection proceedings to take a child into care if the perceived danger in the family home is deemed to be too great. Similarly, in a civil commitment proceeding, a decision has to be made to detain indefinitely a sex offender if the likelihood of committing a further sexual offence is estimated as very high. In both of these instances, one is saying, “ …with these characteristics, I believe that X is so *likely* to occur that one ought to do Y”. The essential point to emphasise here is that in back-casting, an event has already taken place and one’s task is to explain its occurrence while in forecasting, one is predicting the likelihood of an event that might occur in the future. In both situations, however, the expert, when using scientific data, will be faced with the challenge of applying group data to an individual case.

Dawid et al. (7) make a further useful contribution when they distinguish Effect of Causes (EoC) from Causes of Effect (CoE). To illustrate, if one asks the question ‘I have been diagnosed with schizophrenia, how likely is it that I will become violent in the future?’, this would be an instance of an Effect of Causes (EoC) where one could use epidemiological and observational data to answer the likelihood of someone with schizophrenia becoming violent in the future. In contrast, Causes of Effect (CoE) is illustrated by the statement ‘I killed a man a few hours ago and have a diagnosis of schizophrenia. To what extent, if at all, was the killing caused by my schizophrenia?’ The authors make clear in their discussion that CoE is much harder to demonstrate than EoC. This is so, as in experimental terms, this is a singular instance, and would require observing the likelihood of violence in this individual who did and did not have schizophrenia – a logical impossibility. It is important to note that most Court activity concerns CoE.

Hence, Dawid et al. (7), in drawing attention to the expert needing to attend to both the general scientific findings and to the specific causal connections when advancing an opinion on CoE, summarise in stating that ‘…experts’ case-specific conclusions appear to be based largely on an admixture of an unknown combination of knowledge of the subject, experience over the years, commitment to the client or cause, intuition, and blind faith’. Not surprisingly, they conclude that despite such evidence having a scientific veneer, such statements ‘… are little more than educated guesses’ (p. 368).

## 6 The Sally Clark Case – an example of the misreading of scientific evidence and its aftermath

An example of how an expert may mislead the court is provided by the Sally Clark case in the UK. Briefly, after the sudden death one year apart of two of her infant sons, Ms Clark was convicted of their murder and sentenced to life in prison in 1996. Subsequently, it was shown that a medical expert had misinterpreted the statistical evidence which was crucial to her conviction. She was subsequently exonerated on appeal (16).

Due to professional concern raised by this case, the Royal Statistical Society together with the Royal College of Paediatrics and Child Health investigated in a theoretical case study the likelihood of abuse in a child attending an Accident and Emergency Department (A & E). The scenario consisted of an infant presenting with an acute life threatening event together with a nose bleed. It was also revealed that another sibling had died of sudden infant death (SID). The question was whether this presentation and history was sufficient evidence that the child had been abused?

Using Bayesian analysis of conditional probabilities and a systematic review of the literature, the authors concluded that the level of uncertainty in answering this question was much greater than that provided ‘…by expert advice or informal reading of the literature,…’ (17, p. 53). The uncertainty was partly due to the limited evidence from published research.

As in child protection cases, the implications of any decision in either direction are immense with either a child being wrongly taken into care or allowed to stay in the home to suffer further harm. It is small comfort then to learn that the science, as indicated by the Best et al. (17) case study, suggests that currently there is a high level of uncertainty associated with whichever decision is taken due to the absence of good evidence.

## 7 The court’s response to the admissibility of expert evidence

Faigman et al. (6) suggest two strategies whereby the Court can respond to this challenge of applying group evidence to the individual. First, it can restrict the evidence presented by experts to research findings for the group alone (i.e. to framework evidence only). Then it would be for the judge or jury to apply this evidence to the case at hand having been informed by the expert on its general scientific status. The second strategy is to accept expert evidence for both the group and the specific case at hand (i.e., the expert presents both framework and diagnostic evidence or one expert provides framework evidence and another provides diagnostic evidence). This is what many jurisdictions rely on (6). In the Dawid et al. (7) article, a third strategy is described whereby the expert reasons directly from his/her ‘clinical experience’ so that diagnostic evidence is used alone.

Scientific logic would dictate that framework evidence ought to be a necessary condition for the presentation of diagnostic evidence. After all, if there is no framework evidence, then there is no scientific basis for diagnostic evidence. This logic, however, is not always followed in practice. In Zamora v. State (18) for instance, - where the accused tried to argue (unsuccessfully) that watching violent videos was instrumental in causing him to commit a homicide - the Court was presented with both a framework and a diagnostic expert; it excluded the former but allowed the latter. As Dawid et al. (7, p. 365) acidly comment: ‘The Zamora case, in many respects, illustrates a situation that is exactly the opposite of what the model of scientific inference would presume.’ Their comment is supported by some systematic research by Krauss & Scurich (19) who pointed out (p.220) that the Court is more likely to admit as admissible unstructured clinical judgements rather than actuarial-based assessments even though the latter have demonstrable superiority. In another paper Scurich et al. (20) showed that jurors were twice as likely to deny parole to a sexually violent predator (SVP) as compared to another felon even though there was no difference in their likelihood of future violence or further reoffending. The probable reasons for this are outlined further below.

## 8 Implications for forensic mental health

Unfortunately, one has to acknowledge that the research base for much of forensic mental health is weak so that there is likely to be a greater level of uncertainty as compared to other areas of clinical practice (21), (22), (23). As the Best et al. (17) case study illustrates, for instance, the levels of uncertainty surrounding decision-making in child protection proceedings is considerable. Here, the expert witness is faced with two challenges: (a) the absence of adequate data that might underpin framework evidence and (b) a means whereby such framework (or group) evidence – even it were to exist`- can be applied to an individual case.

The papers which we have been citing (especially that of 7) express major reservations on the current status on the science attached to current forensic practice. The question is: are these criticisms valid?

## 9 A proposed answer to criticisms of the current position

Answering these criticisms can be approached at a number of levels and we will proceed to address them as follows: first by describing how the Court assesses expert evidence and how the legal and clinical/scientific approaches differ. Second, that clinical intuition has its own strengths emanating from what is termed ‘tacit knowledge’. Third, by arguing that a Bayesian, rather than a ‘frequentist’ approach, may be a more appropriate way of resolving the difficulty of applying group data to an individual instance, although we acknowledge that this too faces considerable challenges. Finally, how court proceedings might be further strengthened by an adapted epidemiological approach which strengthens clinical causal reasoning.

### 9.1 What the court requires and how legal and clincial/scientific approaches differ

As Faigman et al. (6) point out; there are standards that the Court employs in accepting and evaluating expert evidence. These admissibility criteria rely on the ‘knowledge skills and experience’ of the expert. These, however, are not the only concern of the Court for it must also assess the weight that is attached to the expert testimony (8). This refers to the reasoned argument proffered by an expert witness having regard to all of the facts at his/her disposal; in other words, how plausible is the explanation that is being offered? These authors cite a quotation from Lord Prosser which expresses this well: ‘As the judicial or other opinions, what carries weight is the reasoning, not the conclusion.’ (24). Indeed, it can occur that an expert passes the test of admissibility but the Court subsequently decides that the expert is not competent to express an opinion on a particular issue and even to remove their expert status mid-trial (as occurred in 25, quoted in 8). Here, what appears to be at issue is the plausibility of the expert witness and their capacity to draw on the relevant scientific literature to produce a convincing explanation of a particular event.

Whilst science may question the approach of law, it must accept that the law has its own principles, logic and procedures, which exist to serve quite a different purpose to science. The fact-finding and law-constructing procedures, that may seem odd or cumbersome to some scientists, are rational responses to different constraints that are simply not present in scientific inquiry.

Adjudication in law is a response to a controversy that has to be settled in the here and now. Facts are determined in the theatre of a court trial or inquiry, the method for resolving the dispute must prove satisfactory to the parties, and the results must have public credibility. The events in question cannot be replicated and further observations of what transpired is impossible (26). Scientists, on the other hand, conduct experiments, not a legal trial. The best test of accuracy of scientific data is reproducibility of experiments, their processes and methods, so that replication of observations is its sine qua non. Scientific progress is a gradual process of incremental steps that results in either an acceptance or rejection of a particular hypothesis.

Whilst, both science and law use inductive (probabilistic in nature) logic at fact finding and deductive logic in arriving at conclusions (judgement), the dominant modern perception is that although “the conclusions of legal reasoning commonly are expressed in the deductive form,” and although “[l]ogical validity within this form is often regarded as necessary in legal reasoning,” the deductive form “in itself is of trivial importance”“ (27). It is therefore important to recognize that “reasoning by example’ (hence the importance of illustrative case laws) or analogical reasoning, lies at the centre of the process of legal reasoning. Doing so is to take but the first step toward articulating a satisfactory logic for legal reasoning (28).

### 9.2 Tacit knowledge

The second area that we wish to explore is a particular type of knowledge that clinicians acquire as part of their training. The background to this is is well captured by Best et al. in their discussion of the exercise that they conducted in the aftermath of the Sally Clark case (17). In their response to the discussants of their research (pp. 93-94), while acknowledging that, their analysis quantified the ‘generic chance’ of abuse in children given this history and signs, ‘Extrapolating from this to a particular individual (the ‘to individual’ step) is a very delicate task that depends on many factors including what else the clinician knows about the child and family circumstances’ (p.93) (our italics) In other words, science can take one only so far and it is up to the clinician to integrate all the information at his/her disposal when articulating and defending his/her reasoning. While better science will lead to sounder decision making, science in itself, while being necessary, is insufficient in providing an answer as to how evidence from the group impacts in a particular individual.

If one accepts this argument, there is a need therefore to examine and describe the process of what a clinical expert brings to the table. In this respect Michael Polyani (29) – a philosopher of science - makes a useful contribution when he separates knowledge into that which is either ‘propositional’ or ‘tacit’. The former refers to encoded formulaic knowledge that is exchanged between professionals that is detached from the set of skills required for working in a clinical setting. Tacit knowledge, in contrast comprises skills, ideas and experiences that people have in their minds and are therefore not aware of or how it can be valuable to others. Crucially, the acquisition and effective transfer of this tacit knowledge generally requires extensive personal contact, regular interaction and trust – a situation that is mimicked by the long apprenticeship of clinical training.

Nonetheless, this could leave the expert exposed to the challenge that the current assessment of expert testimony is no further forward than 19^th^ century practitioners whose role has been characterised by Jay (30 p. 17) as ‘Their testimony stressed the weight of their experience – ‘among my 850 patients at Hanwell Asylum’ – and deployed technical language to stress its scientific basis’? Is ‘clinical opinion’ therefore, little more than ‘…a mantra repeated by experts for purposes of legal decision makers, who similarly have little idea what it means’ (7, p. 368).

### 9.3 Bayesian Theory

The third component of our argument considers the place of Bayesian analysis, rather than a frequentist approach, as there has been a move to consider such an approach in resolving the group to individual challenge (see Duggan & Jones (31). Thomas Bayes was an 18^th^ century parish minister who had an interest in conditional probability (i.e. the probability of an event given that some other event had already occurred). Bayes Theorem is a way in which the impact of this new information can be computed. Its benefit is that it allows one to calculate the impact of factors on an individual as opposed to a group. The margin of error is also computed differently in the frequentist and the Bayesian approaches. In the former, the confidence limit defines the upper and lower bounds of an experiment which is repeated many times whereas a Bayesian credible interval gives a direct probability statement (i.e. that the true parameter lies within this specific interval given the data and any prior beliefs).

While this has the advantage of tailoring the risks specific to an individual (rather than to the group), it faces a number of challenges. These include (a) the choice of the prior belief (i.e. the initial belief about the probability of a hypothesis before new evidence is presented) (b) the data may be inadequate to compute the impact of the various variables to arrive at a new posterior belief (i.e. the updated probability of a hypothesis after considering the new evidence). This is a major challenge in forensic mental health as its scientific basis is weak (q.v. above). Finally, there are challenges in relaying to the court the complexity of the calculations and information. Hence, although it has advantages over the frequentist approach, further work is necessary for it to become standard practice in a courtroom setting (32).

## 10 Enhancing scientific rigor with the use of an epidemiologic approach for causal reasoning

While the appeal to ‘tacit knowledge’ as underpinning a plausible explanation provides a partial rebuttal to critics who claim that current practice lacks scientific rigour, we believe that expert evidence could be further enhanced by the incorporation of an approach that adapts an epidemiological approach for causal reasoning. By way of illustration, we present a case which utilises epidemiological principles to anchor expert evidence to a more secure foundation. We consider the causation issue: Here, the task of the expert is to separate the mere a) co-existence of a disorder in a defendant to b) substantial contributory links between the disorder and alleged offence from c) a direct causal link between the mental disorder and the offence, i.e. whether the offence would not have occurred ‘but for’ the presence of the disorder.

To illustrate, consider that for a man diagnosed to suffer from Exhibitionistic Disorder and who exposes his genitals to the distress of the female victim, a framework expert in Exhibitionistic Disorder for example may state that exposing one’s genitals to others is a key feature of this disorder. The ‘theoretical basis’, in this case would be the classificatory systems, e.g. DSM or ICD, which are internationally accepted. The framework expert may then state that by applying the specific diagnostic criteria laid out in these systems, one could make a reliable diagnosis that also may have some validity. Up until this point all of the standards outlined above have been met.

The next step involves expressing an expert opinion on the impact of this disorder on the defendant’s offending. At this point there may be a ‘leap of faith’ from the framework perspective to the opinion that the defendant is not fully criminally responsible, since the offence would not have occurred had he not suffered from Exhibitionistic Disorder. This opinion may amount to a spurious mental health defence since the expert’s opinion does not take into account the legal test implicated, which is whether the defendant knew what he was doing was legally wrong and whether he could control his urges and impulses.

The diagnostic expert’s essential task concerns his or her ability to offer a) a detailed blow by blow subjective account by the defendant about his behaviour and prevailing mental state in the period leading up to and most particularly around the time of the alleged offence, b) gather collaborative evidence from key informants to determine veracity of defendant’s claims about his mental state, c) determine if the defendant’s behaviour around the alleged offence corresponds with objective eyewitness or victim statements and d) if there is incontrovertible forensic and medical evidence that links him to the alleged offence. He is then required to answer the legal questions in the matter. This would usually involve commenting on whether or not his mental disorder may have impacted on his judgement, capacity for rational thinking and impulse control, i.e. his prevailing mens rea at the time. His/her main task would be to filter framework evidence through his or her skill set and experience in order to answer the legal questions for the benefit of the court.

## 11 Proposals for improvement using adapted epidemiologic principles

### 11.1 Inferring causal connections

In law ‘Mens rea’ represents prior guilty mind or ‘intent’ which precedes or is concurrent with ‘Actus reus’ or the physical act of the crime (killing, injuring, stealing, raping, etc.). In this section we suggest ways to determine whether or not and to what extent the ‘mens rea’ (intention) of the defendant, emanating from a diagnosed mental disorder, has a causal relationship with the alleged offence, the ‘actus reus’. We propose considering the following principles adapted from Bradford Hill’s work in evaluating epidemiologic evidence of a causal relationship between a presumed cause and observed effect. (33). This partly stemmed from his work with Richard Doll showing that smoking was associated with the subsequent development of lung cancer (34) Again, we stress that as these are indicators derived from population data rather than individuals, so that the ‘individual step’ requires applying these criteria with clinical expertise using ‘tacit knowledge’ as described above.

#### 11.1.1 Temporality

I.e. the presence of a mental disorder, and thereby its influence on mens rea, must precede the act of omission or commission for which the defendant is charged. Development of a disorder after commission of a crime will not reduce criminal responsibility even if the offender is treated for his mental disorder in a secure hospital.

#### 11.1.2 Dose-response relationship

I.e. the stronger the association between mens rea and actus reus, the more likely it is that there is a cause-and-effect relationship between the two. Here, there are a spectrum of possibilities. (a) There may be no association, i.e. Mens rea is absent as is the case in Sleepwalking or Epilepsy, etc. where the defendant is considered to have been acting like an ‘automaton’ devoid of his own free will. (b) Alternatively, there may be full association between mens rea and actus reus, i.e. the defendant premeditatedly, knowingly and willfully, commits a crime in a clear state of mental reasoning. (c) In other cases, the law recognizes different types of mens rea based upon the strength of the association. Consider the impulsivity of a person with mania who engages in dangerous acts that he would not have done were he mentally well: a mens rea of recklessness. The schizophrenic mother commanded by God’s voice not to feed her infant because she is evil thereby risking the child’s life; a mens rea of gross negligence. Finally, the paranoid man, who speeds in trying to escape imaginary persecutors and is involved in a road traffic accident: a mens rea of blameless inadvertence; these are all examples of varying levels of mens rea associated with mental disorders but not necessarily ‘caused’ by them.

#### 11.1.3 Specificity of association

The degree of specificity of Mens rea that is contributed (partially or entirely) by a mental disorder that leads to an Actus reus is critical in determining the extent of causal link and thereby the level of culpability. There may be a direct or specific link between an abnormal mental state and offence, e.g. a psychotic man who kills someone whilst believing delusionally that he is killing a snake, for example (considered legally insane) or one who has knowledge that he is injuring someone so severely as to cause his death, yet is unable to control his impulse to do so on account of symptoms of his mental disorder thereby reducing the blameworthiness of his actions (considered to have diminished responsibility). In addition having a mental disorder may affect the capacity to form mens rea in a myriad manner which may be relevant to several other defences, e.g. provocation, amnesia, duress, intoxication, etc. It is also quite common to find instances where this relationship does not hold (e.g. someone who is very severely affected by schizophrenia and is not at all violent).

#### 11.1.4 Plausibility

this refers to the underlying reason why a person with a mental disorder may commit an offence. In essence, plausibility refers to underlying motivation. It is plausible for instance for a paedophile who experiences deviant sexual arousal to prepubescent children to cruise around a school (motivation to identify potential victims) that ultimately brings him into contact with a potential victim (intent), whom he eventually abuses sexually (actus reus). It is less plausible for a person with severe depression to be found loitering around a school and who exposes himself to children. The lack of plausibility between a prevailing mental state and the eventual offending act should highlight a greater influence of mental disorder than less. Exploration of this factor to our mind allows an elaboration of the intent (mens rea).

#### 11.1.5 Ruling out alternative explanations

This factor is absolutely critical in distinguishing sincere and reliable experts as opposed to those who will offer the most beneficial ‘causal’ explanation for his/her client, using spurious mental health defenses to exculpate the accused (see 35 for more details). In essence this requires considering various differential diagnoses if the defendant does suffer from a mental disorder and ruling in the one which is most likely. Prior to jumping to conclusions based upon just the subjective account of the defendant, it is essential that the expert takes into consideration the safeguards outlined for a diagnostic expert in the section *‘Tasks of an Expert’.*

#### 11.1.6 The causal explanation fits a legal test

It is essential that the proffered opinion on a putative causal link is relevant to the legal tests at stake in the particular case, e.g. insanity, diminished responsibility, duress, provocation etc. It is even better if there are legal precedents through case law where courts have relied upon certain proposed mechanisms of causality to determine culpability. For instance, if the controversial diagnosis of Battered Woman Syndrome (36, 37) is being used, then the expert must adhere to the exculpatory elements present in a battered woman’s mental state at the material time in order to offer a relevant mental health defence.

### 11.2 Reasonable medical certainty

The concept of ‘reasonable medical certainty’ has been recognized in the US Supreme Court for some time now (Addington v Texas, 441, US 418, 430 (38). The word ‘reasonable’ indicates that there is a range of acceptable solutions to the forensic psychiatric assessment. In contradistinction, the word ‘certainty’ connotes absoluteness (39). So ‘reasonable certainty’ would defy a precise meaning. Notwithstanding the oxymoronic nature of the phrase, individual courts have defined reasonable medical certainty as a threshold starting from just over 50% probability to an upper limit approaching 100%, mirroring the legal standards of proof of preponderance of the evidence (beyond 50%) and beyond a reasonable doubt (nearly 100% certainty) (40). In this connection, it is interesting that Elwood (41) proposes that an offender in Sexually Violent Predator (SVP) evaluation does not meet the criteria for continued commitment unless his risk (more likely than not, or >50%) exceeds the threshold by a credible margin of error. If the credible margin of error were not to exceed 50%, then the individual would not meet the criteria for continued commitment although his individual score was beyond 50%. This approach satisfies Daubert v Merrell Dow Criterion on specifying a margin of error – which was previously mentioned.

## 12 Conclusion

In this paper, we have focused on the critique of current expert evidence in mental health by Faigman et al. (6) and Dawid et al. (7) within a legal context. Although a legal context is limited, we believe that it has relevance to general mental health practice where opinions are sought in a number of different contexts. It therefore brings into focus a number of challenging issues that have relevance in other settings. We acknowledge, however, as a major limitation, that the literature that we have examined is restricted to that from the US/UK and that other jurisdictions may have other processes and criteria.

While both the Faigman et al. (6 and Dawid et al. (7 papers make several important points, we offer a rejoinder that we hope extends the debate and as well as offering alternative interpretations. First, the legal definition of an expert that is relevant to the Court is broader than the strict scientific definition (described in these articles) of an ‘expert’. Second, we argue that the elements of ‘tacit knowledge’ acquired by the expert in his/her training, provides the court with an opinion that is more than an ‘educated guess’ and, in addition, suggest ways in which this can be further strengthened by adopting the epidemiological criteria of Hill (33) in establishing causation.These strategies, we suggest, offer possible solutions to the Group to Individual (G2i) conundrum.
